# Investing in health workers: a retrospective cost analysis of a cohort of return-of-service bursary recipients in Southern Africa

**DOI:** 10.1136/bmjgh-2023-013740

**Published:** 2024-10-07

**Authors:** Sikhumbuzo A Mabunda, Andrea Durbach, Wezile W Chitha, Hawor Phiri, Mahlane Phalane, Sibusiso C Nomatshila, Rohina Joshi, Blake Angell

**Affiliations:** 1 University of New South Wales, Sydney, New South Wales, Australia; 2 Department of Public Health, Walter Sisulu University, Mthatha, South Africa; 3 Law, University of New South Wales—Kensington Campus, Sydney, New South Wales, Australia; 4 Department of Public Health, Walter Sisulu University, Mthatha, Eastern Cape, South Africa; 5 Health, Mpumalanga Department of Health, Mbombela, Mpumalanga, South Africa; 6 Mpumalanga Department of Health, Mbombela, Mpumalanga, South Africa; 7 School of Population Health, University of New South Wales (UNSW), Sydney, New South Wales, Australia; 8 George Institute for Global Health, Delhi, Delhi, India; 9 The George Institute for Global Health, Newtown, New South Wales, Australia

**Keywords:** Health policy, Health systems, Public Health, Cohort study

## Abstract

**ABSTRACT:**

**Background:**

Return-of-service (RoS) schemes are investment strategies that governments use to increase the pool of health professionals through the issuing of bursaries and scholarships to health sciences students in return for service after graduation. Despite using these schemes for many years, Eswatini, South Africa, Botswana and Lesotho have not assessed the costs and return on investment of these schemes. This study aimed to assess the costs and relative rates of contract defaulting in these four Southern African countries.

**Methods:**

A retrospective cohort study was carried out by reviewing databases of RoS beneficiaries for selected health sciences programmes who were funded between 2000 and 2010. Costs of the schemes were assessed by country, degree type and whether bursary holders completed their required service or defaulted on their public service obligations.

**Results:**

Of the 5616 beneficiaries who studied between 1995 and 2019 in the four countries, 1225 (21.8%) beneficiaries from 2/9 South African provinces and Eswatini were presented in the final analysis. Only Eswatini had data on debt recovery or financial repayments. Beneficiaries were mostly medical students and slightly biased towards males. Medical students benefited from 56.7% and 81.3% of the disbursement in Eswatini (~US$2 million) and South Africa (~US$57 million), respectively. Each South African medical student studying in Cuba cost more than five times the rate of medical students who studied in South Africa. Of the total expenditure, 47.7% and 39.3% of the total disbursement is spent on individuals who default the RoS scheme in South Africa and Eswatini, respectively.

**Conclusions:**

RoS schemes in these countries have loss of return on investment due to poor monitoring. The schemes are costly, ineffective and have never been evaluated. There are poor mechanisms for identifying beneficiaries who exit their contracts prematurely and inadequate debt recovery processes.

**WHAT IS ALREADY KNOWN ON THIS TOPIC**

- Return-of-service (RoS) agreements are strategies used to increase the pool of health professionals in many countries and regions including Southern Africa.
- Significant proportions of health budgets are used to fund RoS agreements.
- RoS schemes have never been evaluated to establish their impact and return on investment.
- Some beneficiaries cannot be employed by funding governments due to lack of funds.
- RoS schemes are unidimensional (mostly operate in isolation from other RoS strategies) and poorly coordinated.

**WHAT THIS STUDY ADDS**

- Most data required for monitoring RoS beneficiaries were not available in all four Southern African countries.
- In South Africa, 47.7% of RoS expenditure is spent on individuals who go on to default. In Eswatini, 39.3% of the funding was spent on beneficiaries who went on to default.
- Eswatini had some data on loan recovery for defaulting recipients, South Africa did not have any defaulter reimbursement data.

**HOW THIS STUDY MIGHT AFFECT RESEARCH, PRACTICE OR POLICY**

- To ensure RoS schemes achieve the value for money, they need to be planned, implemented and monitored better.
- Research needs to be an integral part of RoS schemes.
- Policymakers need to overhaul RoS schemes and integrate them with other human resources for health policies.
- Policymakers will give attention to the value of investing on these schemes, that is, the relative value of these schemes compared with other potential uses of finances to train and retain health professionals.

## Introduction

 African health systems face the highest burden of diseases and the lowest density of health professionals.^1^,^5^ This is despite the decades of investment in regulatory, educational and incentive strategies designed to overcome health workforce shortages.^1^,^36 7^ For instance, South Africa, Botswana, Eswatini and Lesotho make use of return-of-service (RoS) schemes to increase the stock of health workers in underserved areas.^3 6^ RoS schemes are conditional scholarships or bursaries which precommit recipients to serve in the health system after completion of their studies.^3 6 8 9^ These schemes provide educational opportunities for children from families with a low socioeconomic status and those living with a disability.^3 6^

In South Africa, RoS schemes are further meant to transform the workforce through gender and racial targeting in favour of females and African (Blacks), respectively. The service requirement is on a year-for-year service basis in the funding province, with no financial repayments (unless they default whereupon they are required to repay the financial support received in full).^3 8 9^ In the other three countries, there are some financial repayment obligations, and beneficiaries are expected to serve up to twice the period of funding support.^6^ In Eswatini for instance, 50% of the funding is a grant and the other 50% has to be repaid within twice the time of funding support if a beneficiary serves the country or repay the full amount if not serving.^6^ In practice, this means that an individual who received 6 years of funding, for example, needs to repay 50% of the total cost of funding within 12 years of completion of their studies if serving the country’s health system.^6^

Each of these schemes is governed by legislation on good audit and accounting practices.^36 10^,^13^ For instance, South Africa’s RoS schemes have to be compliant with the Public Finance Management Act which aims to remove all ‘… fruitless and wasteful expenditures …’.^10^ Similar Acts which also give guidance on the recovery of government funds exist in Eswatini, Botswana and Lesotho.^11^,^13^ A fruitless and wasteful expenditure refers to expenditure that was made in vain, and could have been avoided had proper care been taken.^10^ This study will refer to this type of expenditure as wasteful expenditure.

There is lack of evidence and uncertainty on the effectiveness of these schemes, particularly in Africa.^3 6 14 15^ These schemes were previously found to have a pooled global (most studies were from the USA) contractual fulfilment rate of 71% in 2009.^14 15^ Previous studies have also reported these schemes to have been weakened by structural and systemic challenges which include maladministration, poor coordination between implementing parties, poor record keeping and lack of specificity in the contracts.^3 16 17^

The costs of these schemes are not well understood but they represent a large investment for low resourced health systems.^3 6 18 19^ Furthermore, reductions in health budgets have resulted in the inability of some countries to fill vacant health posts in some situations, regardless of the availability of trained health workers (bursary recipients or others).^18 20 21^ In turn, these health workforce shortages are also associated with increasing public health sector litigations (eg, due to slow response times, fatigue, etc), reducing health budgets further and weakening the ability of health systems to provide for their citizens.^18 20 21^ It is vital to ensure that the scarce resources available to improving population health are used in the most effective way possible.^20 21^ Despite the many years of existence of RoS schemes,^3 6^ neither the cost, impact nor the return on investment of RoS schemes have been assessed in any low-income and middle-income countries (LMICs) or African context. At the very least, RoS schemes should be able to assess the costs of funding and the fulfilment of the year of service, from the payer’s perspective (ie, governments). As previously stated by Mabunda *et al*,^9^ while proposing a RoS scheme conceptual framework, health workforce planning is complex and requires complex solutions. Countries can learn from the labour market analysis framework which focuses on three dimensions, namely, supply and governance of the health workforce, the demand for the health workforce or health services and the population health needs.^19 22^ This study therefore aimed to determine the value of RoS schemes as a worthwhile investment for population health needs in Botswana, Eswatini, Lesotho and South Africa. This study compared the benefits of these schemes to their costs to assess the relative value of investing in these schemes compared with other potential uses of the resources.^23^

## Methods

### Design and participants

A retrospective cohort study was used to assess individuals with complete costing and service records from their admission into an RoS scheme funded at any stage between 2000 and 2010 regardless of when the studies commenced or ended.

### Data collection

**Figure 1:**
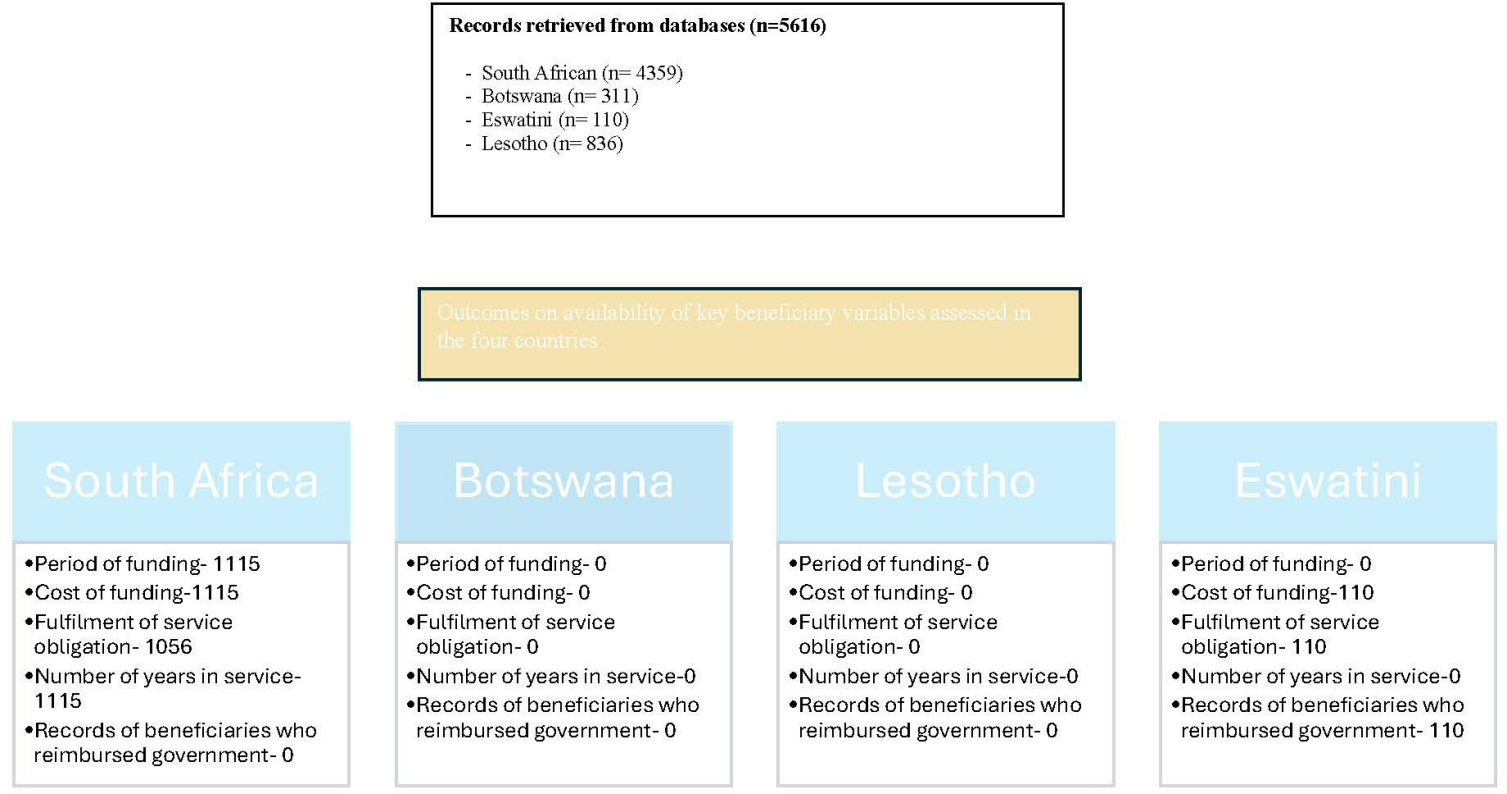
Distribution of beneficiaries and key variable availability in the four countries.

Details of this cohort study are described elsewhere.^8 24^ The latter reference^24^ does not only describe the basic characteristics of all the identified beneficiaries for each of the four countries, but also reports on attrition of beneficiaries and their fulfilment of the service obligation. Briefly, data custodians from South Africa’s nine provincial department of health, the Ministry of Education and Ministry of Labour in in Botswana, the Ministry of Labour and Ministry of Public Service in Eswatini and the National Manpower Development Secretariat (NMDS) in Lesotho were requested for all (not a sample) RoS beneficiary data over the study and service period. Both Botswana and some South African provinces admitted to missing data either in its entirety or in part mostly between 2000 and 2008. A specifically designed access database (online supplemental appendix A) captured data pertaining to demographics, studies (qualification(s) and duration), government expenditures, employment records and their exit from the scheme (eg, death, resignation and retirement). Beneficiaries were limited to seven health degrees namely, medicine, pharmacy, speech therapy, audiology, speech therapy and audiology, occupational therapy and physiotherapy. All but one of the researchers had access only to deidentified data. The one researcher (HP) who had access to identifiable data is an employee of the department of health who has direct access to the South African government payroll system through their employment. This payroll system is indirectly used as a human resources information system. Access to the payroll of the second province (Northern Cape) was led by the provincial RoS custodian. Figure 1 presents a summary of the key variables available from each of the four countries.

### Measurements

#### Exchange rate and adjustment of funding to factor for inflation

Eswatini and South Africa, the two countries with reported data, have an exchange rate of 1:1 South African Rands to Eswatini Lilangeni. South African Rands (ZAR/Rands) were therefore used to report original figures and converted to US dollars (US$) using the fxtop tool historical rates.^25^ US$ rates assessed the value of the US$ on 1 July of the corresponding first year of funding for each beneficiary, for example, an individual whose funding support commenced in 1995 and ended in 2000 would use the US$ exchange rate for 1 July 1995 for the total cost of funding over the 6 years since the data provided were not annualised, but instead provided the total value of the funding support. All costs were adjusted to 2022 ZAR and US$ values using the World Bank GDP deflator.^26^

#### Calculating the pro-rata rates for South African beneficiaries

South Africa calculates the RoS debt due by an individual defaulter pro-rata based on the proportion of time spent in service from the funded period. A prevailing interest is then added to this amount to determine the amount to be reimbursed to government. The equation would then be: Amountdue= [cost-(($\frac{serviceperiod}{fundedperiod})$*cost)+cost*(interest percentage)].

### Data analysis

STATA V.17.0 was used for statistical analyses (Stata Corp., Lakeway Drive USA). Data were summarised using graphs and tables. Proportions were compared using the two-sample test of proportions and a p<0.05 was considered to be statistically significant. The 95% CI was used to reflect the precision of estimates. Results were synthesised using principles of the labour market analysis framework,^22^ namely, the supply (types of supported academic programmes and beneficiary characteristics) and governance of RoS schemes, demand for the health workforce and availability of employment opportunities and the relationship of RoS schemes with population health needs. The STrengthening the Reporting of Observational Studies in Epidemiology (STROBE) checklist (online supplemental appendix B) was used to report the findings.

### Patient and public involvement

Patients and/or the public were not involved in the design or conduct or reporting in this study. However, they should be involved in the dissemination of this study as this policy is not only relevant for health workers and policymakers but also has implications on factors that have an impact on patients’ ability to access healthcare.

### Ethics approval

Ethics approvals were obtained from the University of New South Wales (HC200519), the Botswana Health Research and Development Division (HPDME 13/18/1), the Botswana Ministry of Tertiary Education, Research, Science and Technology (DRST 7/2/13 XXVII (27)), Walter Sisulu University, South Africa (065/2020), the Eswatini Health and Human Research Review Board (FWA 00026661/IRB 000 11253) and the Lesotho Ministry of Health Review Board (ID 242021).

## Results

**Figure 2:**
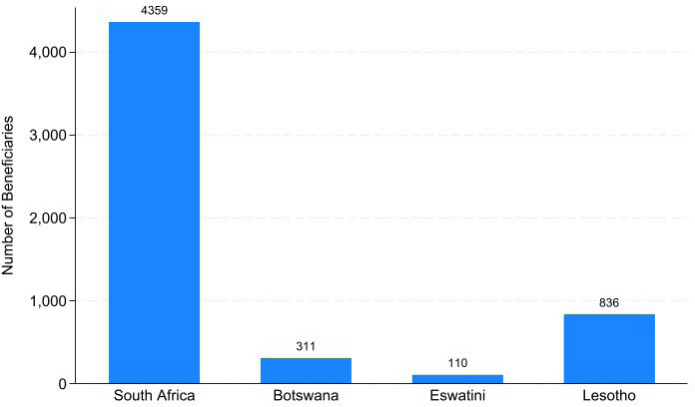
RoS beneficiaries from the four Southern African countries studied. RoS, return of service.

Figure 2 shows that data for 5616 RoS beneficiaries were retrieved from seven of the nine South African provinces (4359), NMDS in Lesotho (836) and the Ministry of Labour in Eswatini (110) and, both ministries in Botswana (1257).

However, of the 5616 beneficiaries, only 19.9% (1115) from two South African provinces (Mpumalanga and Northern Cape) had complete service and costing records, and 110 (2.0%) from Eswatini had partial costing and compliance records. The 1115 individuals had complete service fulfilment records from their admission into the RoS schemes to their service fulfilment until 31 March 2023.

RoS beneficiaries were slightly more likely to be male (50.1%, n=611), mostly studied medicine (60.5%, n=741) and in South Africa (90.1%, n=1104), table 1.

**Table 1:** Beneficiaries’ demographic characteristics

Characteristics	South Africa	Eswatini	Total
	**n=1115 (91.0)**	**n=110 (9.0)**	**n=1225 (100.0)**
Sex; n (%)			
Female	537 (48.2)	74 (67.3)	611 (49.9)
Male	578 (51.8)	36 (32.7)	614 (50.1)
Country of study; n (%)			
Medicine	692 (62.1)	49 (44.6)	741 (60.5)
Pharmacy	195 (17.5)	27 (24.6)	222 (18.1)
Occupational therapy	72 (6.5)	0 (0)	72 (5.9)
Dentistry	73 (6.6)	13 (11.8)	86 (7.0)
Physiotherapy	59 (5.3)	19 (17.3)	78 (6.4)
Speech therapy and/or audiology	24 (2.2)	2 (1.8)	26 (2.1)
Country of study; n (%)			
South Africa	1009 (90.5)	95 (86.4)	1104 (90.1)
Cuba	106 (9.5)	–	106 (8.7)
Lesotho	–	2 (1.8)	2 (0.2)
Russia	–	11 (10.0)	11 (0.9)
Tanzania/Morocco*	–	2 (1.8)	2 (0.2)
Year; n (%)			
1995–2002	228 (20.4)	26 (23.6)	254 (20.7)
2003–2005	264 (23.7)	12 (10.9)	276 (22.5)
2006–2008	360 (32.3)	36 (32.7)	396 (32.3)
2009–2010	263 (23.6)	36 (32.7)	299 (24.4)

* Tanzania and Morocco (one each).

Of the 5.2% (n=58/1115) of South African beneficiaries who failed or died during their studies, 56.9% (n=33/58) were medical beneficiaries, of whom 81.8% (n=27/33) had studied medicine within South Africa (table 2). Overall, 62.5% (n=697/1115) of beneficiaries defaulted their contracts of which 64.3% (n=448/697) were medicine beneficiaries. Overall, one-quarter (n=142/586, 24.2%) of the medicine beneficiaries who had studied within South Africa defaulted outright (without ever serving) and 45.4% (n=266/586) defaulted after fulfilling some of the obligatory service period.

**Table 2:** South African beneficiaries’ progression (1995–2023)

Programmes	Failure of studies/death during studies	Unplanned attritions	Did not serve at all	Served sometime and defaulted	Still serving but not yet served minimum period	Fully served/exceeded	Total
Medicine, South Africa	27 (4.6)*	4 (0.7)†	142 (24.2)	266 (45.4)	38 (6.5)	109 (18.6)	586 (100.0)
Medicine, Cuba	6 (5.7)‡	1 (0.9)§	16 (15.1)	24 (22.6)	53 (50.0)	6 (5.7)	106 (100.0)
Pharmacy	6 (3.1)	1 (0.5)¶	48 (24.6)	73 (37.4)	3 (1.5)	64 (32.8)	195 (100.0)
Dentistry	5 (6.8)	3 (4.1)**	18 (24.7)	17 (23.3)	1 (1.4)	29 (39.7)	73 (100.0)
Physiotherapy	6 (10.2)	0 (0)	16 (27.1)	19 (32.2)	0 (0)	18 (30.5)	59 (100.0)
Occupational therapy	7 (9.7)	0 (0)	25 (34.7)	16 (22.2)	0 (0)	24 (33.3)	72 (100.0)
Speech therapy and/or audiology	1 (4.2)	0 (0)	6 (25.0)	11 (45.8)	0 (0)	6 (25.0)	24 (100.0)
Total	58 (5.2)	9 (0.8)	271 (24.3)	426 (38.2)	95 (8.5)	256 (23.0)	1115 (100.0)

* Deaths during studies=3.

† Death in service=1, ill-health incapacity=3.

‡ Deaths during studies=2; unplanned attritions=death in service, ill-health incapacity, dismissal, relationship breakdown.

§ Fallout with supervisor=1.

¶ Ill-health incapacity=1.

** Death in service=1, dismissal for misconduct=1.

Table 3 shows that while 1.2% of the South African disbursements were spent on audiologists and/or speech therapists, 81.3% was spent on medical students, of which 48.4% was spent on the 15.3% of medical students who studied in Cuba. Each medical student studying in Cuba cost an average of US$260 488 over the duration of their studies, which is 5.2 times the rate of medical students who studied in South Africa. Also shown in table 3, Eswatini disbursed 56.7% of the funding for the education of medical student beneficiaries compared with 1.4% for speech therapy and/or audiology beneficiaries. Defaulters’ whereabouts were unknown, except for two beneficiaries who were unable to find employment.

**Table 3:** Funds disbursed for South African and Eswatini RoS beneficiaries’ studies (1995–2019)

Programmes	Subjects (%)	Total cost (ZAR)	Total cost in US$ (%)	Average cost/beneficiary (US$)
	**South Africa**			
Medicine, South Africa	586 (52.6)	212 433 972	29 453 941 (41.9)	50 263
Medicine, Cuba	106 (9.5)	206 989 809	27 611 738 (39.3)	260 488
Pharmacy	195 (17.4)	42 678 671	5 824 444 (8.3)	29 869
Dentistry	73 (6.5)	18 464 006	2 576 573 (3.7)	35 296
Physiotherapy	59 (5.3)	12 506 683	1 696 219 (2.4)	28 759
Occupational therapy	72 (6.5)	16 597 842	2 256 367 (3.2)	31 338
Speech therapy and/or audiology	24 (2.2)	6 078 408	810 285 (1.2)	33 762
Total	1115 (100.0)	515 749 391	70 229 567 (100.0)	62 986
	**Eswatini**			
Medicine	49 (44.5)	15 288 010	2 014 182 (56.7)	41 106
Pharmacy	27 (24.5)	4 632 185	597 940 (16.8)	22 146
Dentistry	13 (11.8)	3 969 268	524 833 (14.8)	40 372
Physiotherapy	19 (17.3)	2 742 835	363 627 (10.2)	19 138
Speech therapy and/or audiology	2 (1.8)	392 343	51 083 (1.4)	25 542
Total	110 (100.0)	27 024 641	3 551 665 (100.0)	32 288

RoS, return of service.

Of the total expenditure by the two South African provinces, over US$33 million (47.8% of total disbursement) was spent on individuals who went on to default (table 4). Defaulting medicine beneficiaries were responsible for over US$26 million of this loss (79.3%). The total invested on defaulters is US$18 571 458 and 14 992 786 for those who default outright and those who defaulted after serving some time, respectively. Only 1.3% (US$434 262) and 3.8% of this deficit is attributable to speech therapy and/or audiology and occupational therapy beneficiaries, respectively.

**Table 4:** Expenses disbursed on defaulting South African RoS beneficiaries

Programmes	Individuals who did not serve at all		Individuals served some time*		All defaulters	
	N (%)	Cost (US$)	N (%)	Cost (US$)	N (%)	Cost (US$)
Medicine, South Africa	173 (51.8)	8 579 974	266 (62.0)	8 329 120	439 (57.5)	16 909 094
Medicine, Cuba	23 (6.9)	5 603 900	24 (5.6)	4 113 131	47 (6.2)	9 717 031
Pharmacy	54 (16.2)	1 681 301	73 (17.0)	1 317 899	127 (16.6)	2 999 200
Dentistry	23 (6.9)	834 098	20 (4.7)	439 725	43 (5.6)	1 273 823
Physiotherapy	22 (6.6)	604 091	19 (4.4)	345 867	41 (5.4)	949 958
Occupational therapy	32 (9.6)	1 014 241	16 (3.7)	266 635	48 (6.3)	1 280 876
Speech therapy and/or audiology	7 (2.1)	253 853	11 (2.6)	180 409	18 (2.4)	434 262
Total	334 (100.0)	18 571 458	429 (100.0)	14 992 786	763 (100.0)	33 564 244

* Costs for individuals who served are calculated pro-rata based on the period of service already given, US$.

RoS, return of service; US$, US dollar.

**Figure 3:**
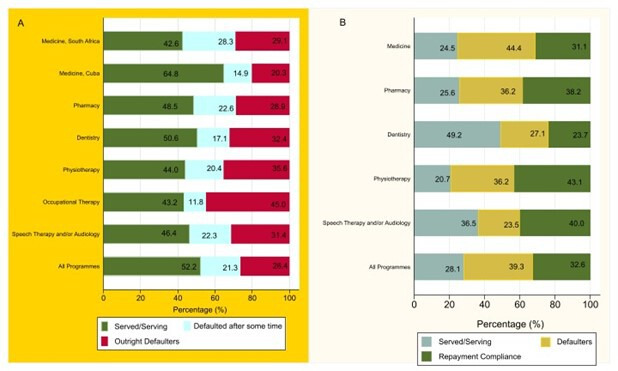
Proportional expenditure of South African RoS schemes on defaulters (**A**) and proportional RoS loan component owed by defaulters and non-defaulters in Eswatini (**B**). RoS, return of service.

Figure 3A also shows that 57.4% of funding spent on medical students who studied within South Africa was associated with those who defaulted their bond. However, only 35.2% of funding spent on South African medical students who studied in Cuba was associated with those who defaulted their bonds, and these differences were statistically significant (p<0.0001). Other notable wasteful expenditures spent on defaulters are for occupational therapy (56.8%), physiotherapy (56.0%), speech therapy and/or audiology (53.7%) and pharmacy (51.5%). For South Africa, this suggested that for every US$100 invested in RoS schemes, US$47.7 were lost through defaulting of the scheme. Eswatini expects all beneficiaries to repay 50% of the funding if serving and 100% if not serving. In Eswatini, defaulting medicine beneficiaries owed 44.4% of the disbursement for medical studies, followed by 36.2% for pharmacy and physiotherapy beneficiaries (figure 3B) and table 5. Overall, only 32.6% of Eswatini’s beneficiaries had fulfilled their repayment terms regardless of the defaulting status. Also noted is that 39.3% of the amount due in Eswatini was owed by defaulting beneficiaries.

**Table 5:** RoS disbursement and loan recovery in Eswatini (2000–2023)

Programmes	Owed by defaulters			Owed by those serving		
	N (%)	Cost (US$)	Average owing (US$)	N (%)	Total cost (US$)	Average owing (US$)
Medicine	31 (63.3)*	893 717	28 830	18 (36.7)	493 322	27 407
Pharmacy	15 (55.6)	216 451	14 430	12 (44.4)	152 868	12 739
Dentistry	4 (30.8)†	142 023	35 506	9 (69.2)	258 236	28 693
Physiotherapy	12 (63.2)	131 595	10 966	7 (36.8)	75 378	10 768
Speech therapy and/or audiology	1 (50.0)	12 027	12 027	1 (50.0)	18 623	18 623
Total	63 (57.3)	1 395 813	22 156	47 (42.7)	998 427	21 243

* Includes two unemployed beneficiaries.

† Includes one beneficiary who died during studies.

RoS, return of service.

While 23.0% (257/1115) of South African beneficiaries fulfilled their contractual obligation, it was impossible to determine the return on investment of 95 South African beneficiaries as they were in varying stages of their contractual fulfilment but had not yet met the minimum obligatory period. Compared with beneficiaries who were in varying stages of their service fulfilment, South African beneficiaries who had served or exceeded their service obligation comprised of 74.1% (109/147) medical students who studied within South Africa, 10.2% (6/59) medical students who studied in Cuba, 95.6% (65/68) pharmacists, 96.7% (29/30) dentists and 100.0% of physiotherapists (n=18), occupational therapists (n=24) and speech therapists and/or audiologists (n=6), respectively.

## Discussion

This cohort study aimed to ascertain the relative costs and level of defaulting on contracts by beneficiaries of ROS schemes in four Southern African countries. Most nations did not have complete cost and service data to report. Only two South African provinces and Eswatini had some relevant cost data. Eswatini was the only country that had information on debt/loan recovery. Medicine beneficiaries received 81.3% of the funding in South Africa and 56.7% in Eswatini. A medical student studying in Cuba cost South Africa’s economy five times more than those studying within the country. More than half (57.4%) of the expenditure on South African medical beneficiaries who had studied within the country was spent on those who went on to default compared with 35.2% for those who studied in Cuba. Only 5.7% of those South Africans who studied in Cuba had fulfilled their service obligations at the time of the study compared with the 18.6% who had studied locally, though 50% were still meeting their obligations but not yet fulfilled them. The study further found that for every US$100 investment into RoS schemes in South Africa, about US$48 was devoted to workers who subsequently defaulted on their contracts. This loss is in addition to the loss of human capital associated with losing a skilled-health worker in these resource-constrained health systems. While it is commendable that Eswatini had debt recovery data, 39.3% of the debt was owed by defaulters whose whereabouts were largely unknown. This therefore makes it unlikely that the funds would be recovered.

This study found the schemes to be ineffective, costly and an ineffective and costly means to improve the stock of skilled health professionals in these systems. Our analysis demonstrates that the expenditure being directed to these schemes was large, exceeding South Africa’s Mpumalanga province’s budget for emergency medical services by 5.6% in 2023, and accounting for the equivalent of 15.3% of the referral hospital budget and 5.1% of the District Health Services’ (Primary Healthcare) budget for 2023. In addition, the RoS allocation for the two South African provinces (88.9% of which are from Mpumalanga), is 17.2%, 23.9% and 26.4% of Mpumalanga province’s Ehlanzeni, Gert Sibande and Nkangala health district budgets for 2023, respectively.^27^ There is limited evidence that this is an effective use of scarce health system funds. Almost a half of this initial disbursement (47.7%) is directed to training health workers who do not meet their public sector service obligations and is not recovered. Internal mechanisms are unable to detect individuals who exit the system before completing their obligatory service commitments. These have dire consequences for health systems of a country in the global South. Implicitly, poor monitoring of beneficiaries’ compliance could support other findings of weak support structures for beneficiaries who might have professional and personal challenges in their placement sites, thus resulting in frustration and premature departure.^1 2 16 17^ As of January 2022, National Health Service Corps (NHSC) funded some beneficiaries in their final year of training (eg, medical students, dental students, nurse practitioners, etc) US$120 000 for a 3 year service commitment in an approved primary care facility.^28^ This expense by a high-income country is comparable to that spent by South Africa on Cuban studying beneficiaries (~US$260 488) for an expected 8–10 years of service in a poorly defined location.^28^

The phenomenon of failure to retrieve data is not unique to this study and has been observed in other settings, including high income programmes such as the NHSC, however, cannot be justified.^29^ Furthermore, even though defaulters are meant to reimburse government for defaulting and/or a loan component in the other countries, data on amounts recovered were not available in any of the South African provinces, Botswana or Lesotho for the study period, potentially further perpetuating the wastage. All these factors therefore result in audit disclaimers due to poor data quality and wasteful expenditures.^10 30^ The fact that 30.0% of South African RoS beneficiaries could default outright without any consequences demonstrates that the investment is not well monitored.

Even though the Cuban medical programme was a political programme that arose out of need due to shortages of medical schools within South Africa, there is no evidence to justify spending five times more to educate a medical doctor as you would in South Africa.^31^,^41^ If anything, the programme has not proven to be any more effective as only 5.7% of RoS beneficiaries who studied in Cuba have ever completed or exceeded their service obligations in the two South African provinces with completed data. Previous descriptive studies had focused on the location of these Cuban trained beneficiaries and, not the fulfilment of their service obligations and the cost of the programme.^31^,^41^ Some or most of the amount spent on this programme could likely be better directed towards the increase of the country’s internal capacity to train health sciences students, better support and retain health workers through other mechanisms and to further strengthen the entire health system.^1^ Furthermore, other alternatives include the investment on task-shifting strategies such as the investment on mid-level cadres like clinical associates, dental therapists, occupational therapy assistants, physiotherapy assistants, etc., for some activities.^42^,^48^

Allocation of funding to different programmes is biased towards medical beneficiaries and only a few speech therapists and/or audiologists benefit from the schemes. Previous qualitative studies in the four countries under study have suggested that the funded programmes are not evidence-based, are biased towards medicine and the future service needs are poorly defined.^3 6^ Future studies could investigate the unintended consequences of this poor planning. Through the labour market analysis framework, it is evident that merely training of the health workforce is not sufficient for return to service.^19 22^ The relative value of RoS schemes in increasing the pool of available health workers will ultimately depend on whether there are more effective policy instruments/tools to achieve this aim within available resources. It is vital, however, that all such policy interventions are subject to rigorous evaluation of their ongoing cost and impact on the health workforce.

It is also important to explore the impact of programmes that support the health professionals once in service, as the reasons for the premature termination are not known. Such programmes could include making the conditions for these beneficiaries more reasonable once they graduate as this could be a better use of money and reduce the outflow of health workers who are already trained. A similar suggestion has previously been made by Rabinowitz *et al*^29^ in the American context who proposed targeted admission, improved curricular experiences and support for those likely to fulfil their service commitment.^29^ It is also important to undertake an in-depth analysis of the beneficiaries who successfully met their obligations as these schemes could easily be better targeted to those who are more likely to serve their contracts. Benefits of these are seen with the Jichi Medical University’s (JMU) programme in Japan where all graduates have a rural obligatory service period.^49^ The programme has a retention rate of 97% of its beneficiaries.^49^ For instance, the JMU’s programme previously associated lower retention with private high school attendance, and mothers with a higher education background than those who were compliant.^49^ Rural upbringing, early exposure to rural practice environments and mentorship are also associated with higher retention or intention to practice in rural practice environmnents.^12 29 50^,^52^

This research has shown the importance of evidence based planning and timely evaluation of programmes. Had there been evaluation plans and actual evaluations most of these wastages could have been mitigated. To the researchers’ knowledge, this is the first study evaluating the economic returns/losses of RoS schemes in LMICs. In the absence of precedent studies and data limitations, there were several limitations to our work. The US$ exchange rate used is for the beginning of the study period and not the actual exchange rate at the time of the disbursement. It is hoped that the rates will even out as they were applied consistently from 1995 until 2010. Missing data did not only limit the ability to ascertain the true costs of running these schemes, but it also limited the ability to determine representativeness of the data. Notwithstanding, the study has demonstrated high costs of RoS schemes, inequitable allocation of funds to programmes, wasteful expenditures with lack of financial recovery mechanisms and high loss of human capital investments to the funding province’s public sector.

## Conclusions

Despite longstanding use of RoS schemes in Botswana, Eswatini, Lesotho and South Africa, and substantial amounts invested, the schemes have not been proven to be an effective investment to improve the health workforce stock in these nations. These schemes are ineffective, are costly, have high defaulter rates and are biased towards medical beneficiaries. Almost half the amount invested in these programmes is effectively lost to the public system, and these losses are compounded when the valuable human capital represented by scarce health workers is also considered. The governments of these countries need to build internal training capacity to minimise the exorbitant fees spent on international programmes which can be five times more costly than local programmes. Governments need to further ensure that investments into RoS schemes represent value for money, which likely requires improvements to the effectiveness of these schemes through the strengthening of monitoring and evaluation, and improved planning. Planning and allocation decisions taken through these schemes should be based on evidence to minimise waste and maximise the impact on population health that these investments can have across these nations.

## Supplementary material

10.1136/bmjgh-2023-013740 online supplemental file 1

10.1136/bmjgh-2023-013740 online supplemental file 2

## Data Availability

Data are available upon reasonable request.
