# Supplementary material for: Investing in health workers: a retrospective cost analysis of a cohort of return-of-service bursary recipients in Southern Africa
Source: BMJ Glob Health. 2024 Oct 7;9(10):e013740. doi: 10.1136/bmjgh-2023-013740 (PMC11459328; doi:10.1136/bmjgh-2023-013740)

**Subject Number**	0	**Auto Number**	1
Country			
Province/Region		Mother Alive	
		Father Alive	
		Primary Carer	
Gender		Primary Carer employed	
Race		Both Parents employed	
Date of Birth		Household Source of Income	

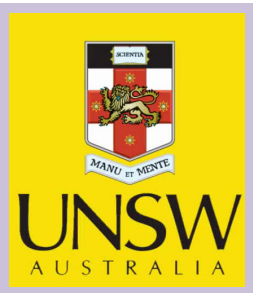

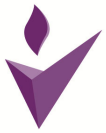

The George I
for Global Health

nstitute

Employer 7 Name	
Employer 7 Date of Commence	

Postal Code (at Application)

Household income amount per annum (Rands/Pula)

School Postal Code

Name of High school

Additional Notes on Bursary Contract

Year of Matriculation

Year of A or B levels

Aggregate Results

Additional Notes on any prior learning

SUBJECT	RESULTS
Mathematics	
Physical Science	
Life Orientation	
Life Sciences/Biology	

Marital Status at Completion of studies

Date of Commencement of Internship

Internship hospital 1 (Name)

Internship hospital 1 (Date of departure)

Internship hospital 2 (Name)

Internship hospital 2 (Date of Commencement)

Internship hospital 3 (Name)

Internship hospital 3 (Date of Commencement)

Date of Completion of internship

Date of commencement of Community service

Community Service Hospital1 (Name)

Community Service Hospital1 (Date of Departure)

Employer 7 Job Title

Employer 7 Date of Departure

Employer 8 Name

Employer 8 Date of Commence

Employer 8 Job Title

Employer 8 Date of Departure

Employer 9 Name

Employer 9 Date of Commence

Employer 9 Job Title

Employer 9 Date of Departure

Employer 10 Name

Employer 10 Date of Commenc

Employer 10 Job Title

Employer 10 Date of Departure

Name of University	
Date when bursary offer was made	
Year of first enrolment for Academic Program	
Marital Status at Commencement of studies	
Secondary University of Study if Applicable	
Additional Information on Tertiary Studies	
Presence of bursary offer contract signed by all parties (Year1)	
Presence of bursary renewal contract signed by all parties Yr2	

Country of Study	
Academic Year of Study when bursary was issued	
Academic Program of Study	
Did beneficiary complete their studies	
Year of completion of studies	
Presence of bursary renewal contract signed by all parties Yr7	
Presence of bursary renewal contract signed by all parties Yr8	
Post completion service area specified (Yes/No)	
Name of Post completion service area	
Cost of Sponsorship (ZAR/Pula) Year1	
Cost of Sponsorship (ZAR/Pula) Year2	

Community Service Hospital2 (Name)	
Community Service Hospital2 (Date of Commencement)	
Date of Completion of Community Service	
Employer 1 Name	
Employer 1 Date of Commencement	
Employer 1 Job Title	
Employer 1 Date of Departure	
Employer 2 Name	
Employer 2 Date of Commencement	
Employer 2 Job Title	
Employer 2 Date of Departure	
Employer 3 Name	
Employer 3 Date of Commencement	
Employer 3 Job Title	
Employer 3 Date of Departure	
Employer 4 Name	
Employer 4 Date of Commencement	
Employer 4 Job Title	
Employer 4 Date of Departure	

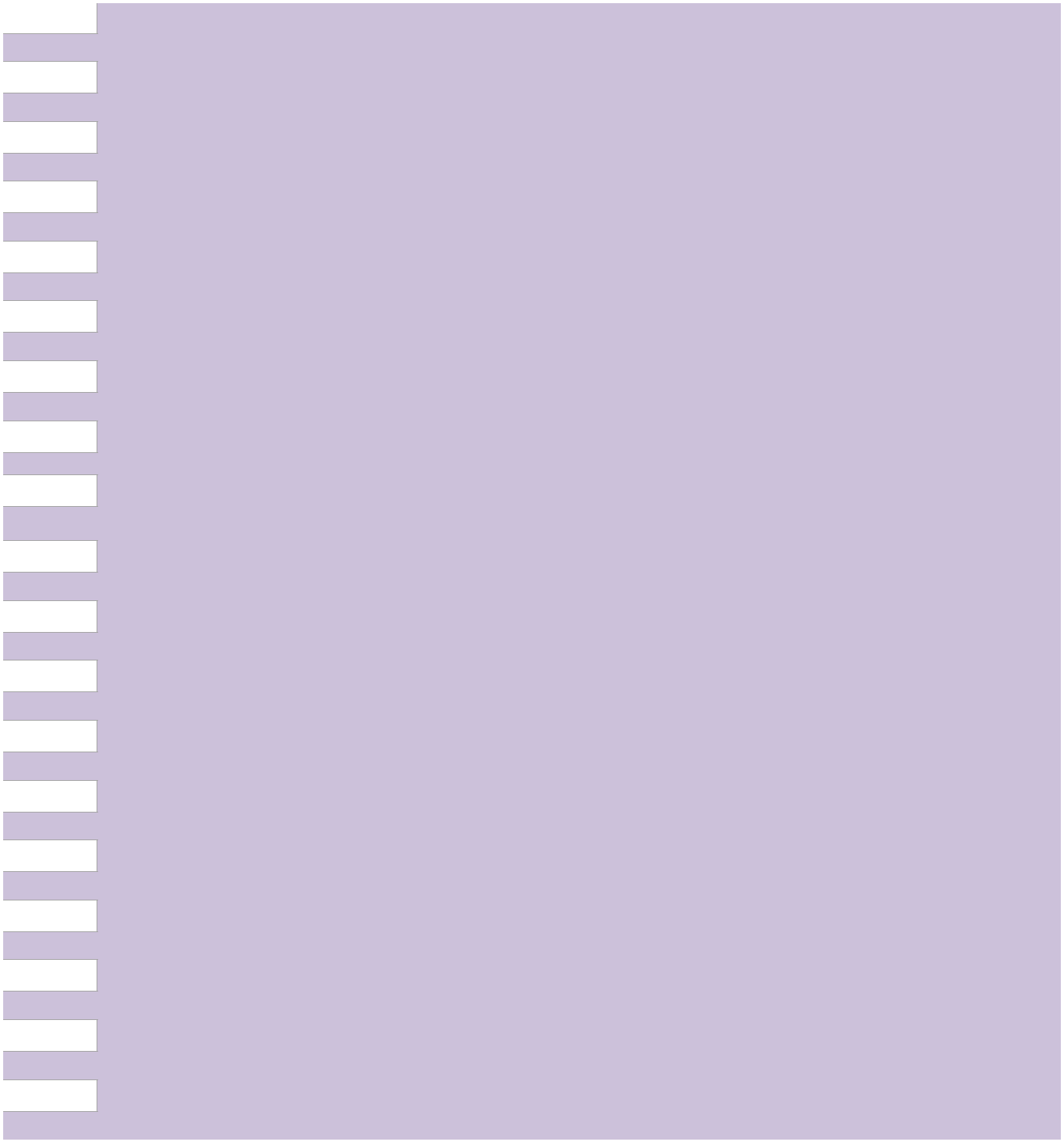

Presence of bursary renewal contract signed by all parties Yr3		Cost of Sponsorship (ZAR/Pula) Year3		Employer 5 Name	
				Employer 5 Date of Commencement	
		Cost of Sponsorship (ZAR/Pula) Year4			
Presence of bursary renewal contract signed by all parties Yr4				Employer 5 Job Title	
		Cost of Sponsorship (ZAR/Pula) Year5		Employer 5 Date of Departure	
				Employer 6 Name	
Presence of bursary renewal contract signed by all parties Yr5		Cost of Sponsorship (ZAR/Pula) Year6			
				Employer 6 Date of Commencement	
		Cost of Sponsorship (ZAR/Pula) Year7			
Presence of bursary renewal contract signed by all parties Yr6				Employer 6 Job Title	
		Cost of Sponsorship (ZAR/Pula) Year8		Employer 6 Date of Departure	

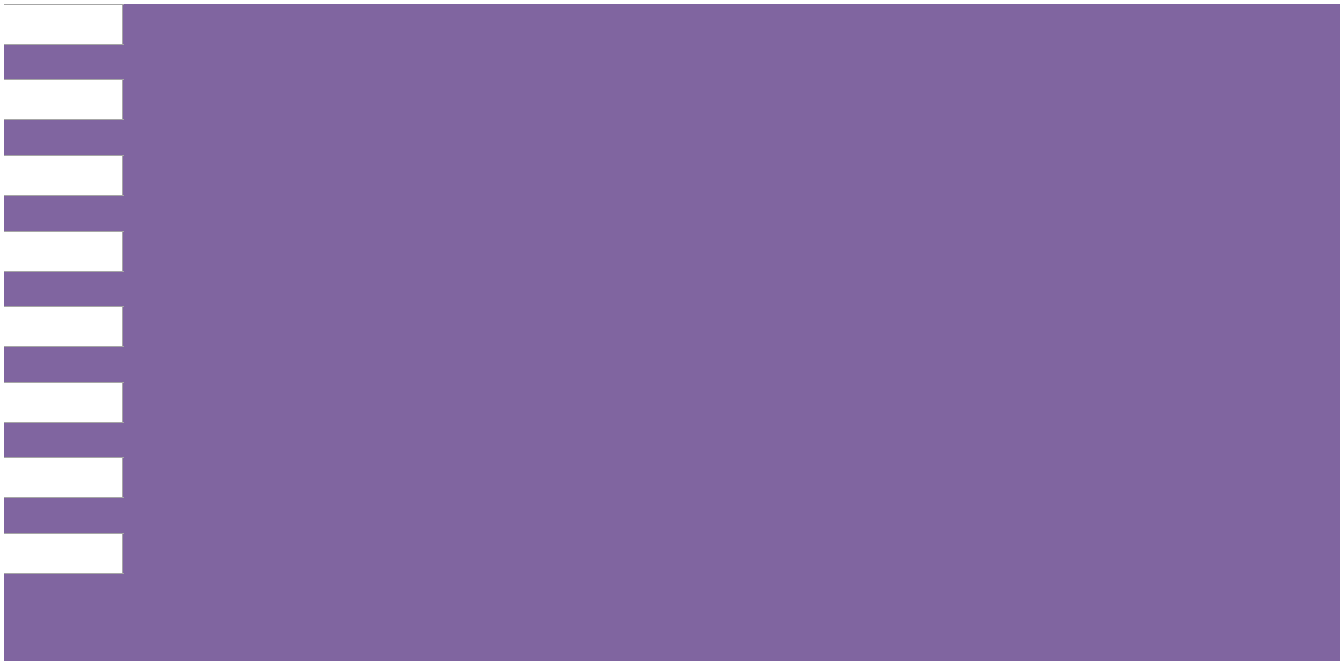

Supplement: online supplemental file 1 [file bmjgh-9-10-s001.pdf]
